# Genes, Cognition, and Their Interplay in Methamphetamine Use Disorder

**DOI:** 10.3390/biom15020306

**Published:** 2025-02-19

**Authors:** Ramisha Khan, Alyna Turner, Michael Berk, Ken Walder, Susan Rossell, Alexandre A. Guerin, Jee Hyun Kim

**Affiliations:** 1 IMPACT, Institute for Innovation in Physical and Mental Health and Clinical Translation, School of Medicine, Deakin University, Geelong, VIC 3220, Australia; s223324846@deakin.edu.au (R.K.); a.turner@deakin.edu.au (A.T.); michael.berk@deakin.edu.au (M.B.); ken.walder@deakin.edu.au (K.W.);; 2 Centre for Mental Health, Swinburne University of Technology, Melbourne, VIC 3122, Australia; srossell@swin.edu.au; 3 Centre for Youth Mental Health, University of Melbourne, Melbourne, VIC 3010, Australia; alexandre.guerin@unimelb.edu.au; 4 Orygen, Melbourne, VIC 3052, Australia

**Keywords:** *BDNF*, *FAAH*, *SLC18A1*, *SLC18A2*, cue reactivity, skin conductance response, neurobiology, neuroinflammation, inhibitory control, Stroop performance

## Abstract

Methamphetamine use disorder is a pressing global health issue, often accompanied by significant cognitive deficits that impair daily functioning and quality of life and complicate treatment. Emerging evidence highlights the potential role of genetic factors in methamphetamine use disorder, particularly in association with cognitive function. This review examines the key genetic and cognitive dimensions and their interplay in methamphetamine use disorder. There is converging evidence from several studies that genetic polymorphisms in *BDNF*, *FAAH*, *SLC18A1*, and *SLC18A2* are associated with protection against or susceptibility to the disorder. In addition, people with methamphetamine use disorder consistently displayed impairments in cognitive flexibility and inhibitory control compared with people without the disorder. These cognitive domains were associated with reactivity to methamphetamine cues that were positively correlated with total years of methamphetamine use history. Emerging research also suggests that inhibitory control is negatively correlated with lower blood *FAAH* mRNA levels, while cognitive flexibility positively correlates with higher blood *SLC18A2* mRNA levels, highlighting how genetic and cognitive dimensions interact in methamphetamine use disorder. We also include some future directions, emphasizing potential personalized therapeutic strategies that integrate genetic and cognitive insights. By drawing attention to the interplay between genes and cognition, we hope to advance our understanding of methamphetamine use disorder and inform the development of targeted interventions.

## 1. Introduction

Amphetamine-type stimulants are among the most widely used illicit substances in the world [1], primarily due to their low cost, easy accessibility, and dramatic subjective effects that have contributed to the significant global rise in methamphetamine consumption [2]. In particular, methamphetamine use is emerging as a crisis in Australia, being the most consumed illicit substance across all regions [3] and costing AUD 10.58 billion annually [4].

Methamphetamine is a psychostimulant that affects the central nervous system through multiple mechanisms. The acute effects of methamphetamine use include feelings of well-being, euphoria, alertness, and increased libido [5]. However, chronic use can lead to harmful consequences, such as increased blood pressure, muscle tremors, insomnia, anxiety, hyperthermia, stroke, stomach cramps, psychotic symptoms like paranoia and hallucinations, and ultimately, methamphetamine use disorder [6]. A number of methamphetamine effects can be attributed to neurotoxicity, which can result in loss of grey matter, a smaller hippocampus, an enlarged striatum, and altered vasculature, along with other morphological changes contributing toward methamphetamine use disorder [7].

The current treatment strategies for methamphetamine use disorder focus on addressing methamphetamine-induced neurotoxicity [8] and behavioral therapies [9]. However, 61% of people with methamphetamine use disorder who complete treatment relapse within one year, and a further 25% relapse within five years [10]. Treatment efficacy may be limited without considering the critical role of cognition [11,12], with reduced inhibitory control associated with cue-induced craving in people with methamphetamine use disorder [13], suggesting a role for cognition in relapse.

In addition, methamphetamine use disorder involves genetic predispositions [14,15,16]. For example, genetic studies including genome-wide association studies (GWASs) [17,18,19,20] and a meta-analysis [14] have identified specific genetic variants linked to the risk of methamphetamine use disorder. In the present review, we will discuss the genetic and cognitive factors and their potential interplay in people with methamphetamine use disorder to understand ways to improve treatment outcomes. It is important to note that this review focuses on human studies with clear clinical implications. While there are a few very recent epigenetic studies on people who use methamphetamine [21,22,23], there is yet poor converging evidence and no link to cognition, hence covering epigenetics is beyond the scope of this review.

## 2. Neurobiology of Methamphetamine Use: A Summary

**Figure 1:**
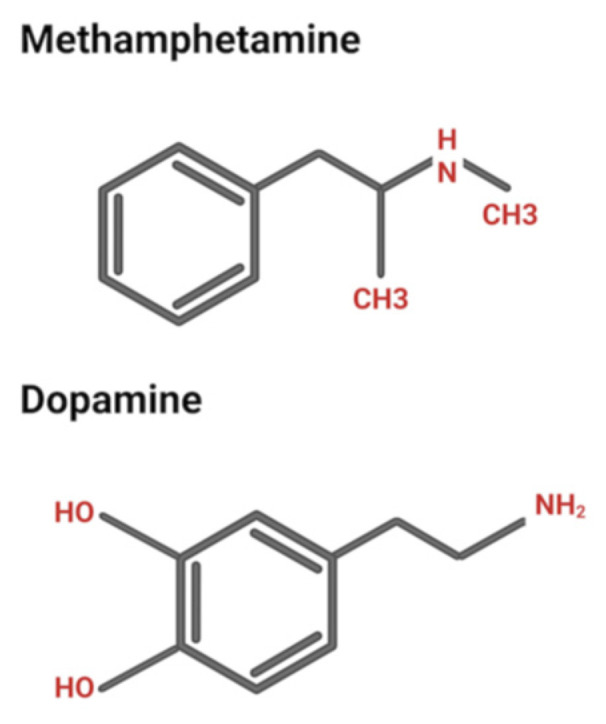
Chemical structures of methamphetamine and dopamine.

Methamphetamine is a potent drug that can easily cross the blood–brain barrier and the cell membrane aided by its lipophilic nature. Methamphetamine shares structural similarity with dopamine (Figure 1), allowing it to be taken up by dopamine transporters (DATs) into dopaminergic neurons, where it exerts potentially neurotoxic effects [24]. Methamphetamine reduces the number of available dopamine transporters on the cell surface, resulting in decreased reuptake of dopamine and a subsequent accumulation of extracellular dopamine [25,26]. This increase in dopamine concentration contributes to feelings of methamphetamine reward that lead to its use and seeking [24]. Methamphetamine also inhibits and reverses the serotonin and norepinephrine transporters on the cell surface [27,28,29], leading to an increase in serotonin and norepinephrine levels in the synapses [30,31]. Elevated serotonin contributes to the mood-related effects of methamphetamine [32], while increased norepinephrine levels are associated with the stimulant effects of methamphetamine, such as heightened heart rate and blood pressure [33].

**Figure 2:**
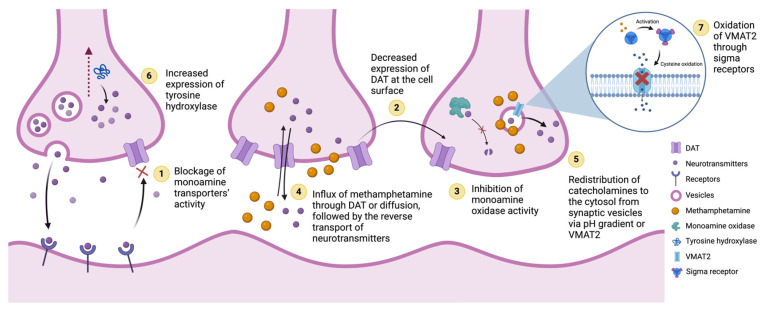
Mechanisms of methamphetamine-induced dopamine release. (1) Inhibition of dopamine reuptake by the blockage of dopamine transporter (DAT) activity [53]. (2) Decreased expression of available cell-surface DATs [25]. (3) Inhibition of monoamine oxidase activity that leads to increased dopamine, serotonin, and norepinephrine levels [54]. (4) Influx of methamphetamine through DAT or plasmalemma diffusion, followed by the reverse transport of neurotransmitters [55]. (5) Redistribution of monoamines from synaptic vesicles to the cytosol via methamphetamine changing the pH gradient and/or binding to VMAT2, increasing the cytosolic levels of dopamine [48,49,50,56]. (6) Increased expression of tyrosine hydroxylase, which helps in the production of dopamine, thereby increasing dopamine levels [57]. (7) Activation of sigma receptors, which in turn causes the oxidation of vesicular monoamine transporter 2 (VMAT2), ultimately affecting the transporters’ function [58].

The release of neurotransmitters is achieved via several different mechanisms, summarized in Figure 2. These coordinated interactions with multiple monoamine transporters not only produce the immediate stimulant effects of methamphetamine but also contribute to long-term neurobiological changes [34,35,36]. Intracellular methamphetamine, whether DAT-mediated or diffused through the plasmalemma, binds to vesicular monoamine transporter 2 (VMAT2) and redistributes it to reduce it at the synapse and in the vesicles [37,38,39,40,41,42,43]. VMATs are responsible for loading monoamines into intracellular vesicles for storage [44,45,46,47]. The methamphetamine-related decrease in synaptic VMAT2 is associated with a reduced number of intracellular vesicles to sequester dopamine [37,38,39]. Furthermore, methamphetamine is a weak base similar to amphetamine, leading to a significant efflux of monoamines out of the intracellular vesicles due to the pH gradient [48,49,50]. These processes initially lead to excess dopamine in the cytosol, which contributes to neurotoxicity, neuroinflammation, mitochondrial dysfunction, and resultant oxidative stress caused by methamphetamine [51]. With chronic methamphetamine use, however, neurotoxicity, neuroinflammation, and oxidative stress can reduce basal monoamine levels in the nerve terminals [52].

Neuroinflammation is mediated by inflammatory changes in microglia, a process known as microgliosis, which is an early response to methamphetamine use but can persist for at least 2 years [59] even after abstinence [60]. The pharmacological profile of methamphetamine reveals that its ability to activate microglia is indicative of neurotoxicity rather than merely a response to damage at nerve terminals [61]. Methamphetamine induces neuroinflammation by activating innate immune toll-like receptor 4 (TLR4) along with its coreceptor myeloid differentiation protein 2 (MD-2), which stabilizes the heterotetramer (TLR4/MD-2)_2_, thereby enhancing the expression of markers associated with microglial activation [62]. Beyond TLR4, methamphetamine is linked with the activation of the nucleotide oligomerization domain-like receptor protein 3 inflammasome in microglia, leading to increased release of interleukin-1β [63], which plays a key role in the orchestration of central nervous system inflammation and contributes to the risk of developing drug dependence [64]. Inflammasome-mediated interleukin 1β maturation and secretion, in turn, mediates microglia-induced neurotoxic [63] and excitotoxic effects [65], a process that is also evident in several conditions, including neurodevelopmental and neurodegenerative disorders [66].

In a broader context, methamphetamine’s neurotoxic effects have been hypothesized to stem from several interdependent mechanisms, which include the following: (1) excessive dopamine release, leading to increased production of reactive oxygen species, such as peroxides, which can adversely affect cell structures; (2) ubiquitin–proteasome system dysfunction, leading to autophagy resulting from the activation of the intracellular degradation system; (3) protein nitration, resulting in increased levels of the free radical nitric oxide, which can cause cytotoxicity; (4) endoplasmic reticulum stress, triggering apoptosis; (5) increased expression of p53, arresting cell cycles and altering DNA repair; (6) increased levels of inflammatory cytokines, leading to neuroinflammation; (7) dopamine D_3_ receptor activation, resulting in methamphetamine-induced hyperthermia; and (8) microtubule deacetylation, damaging the blood–brain barrier [67]. Additionally, excitotoxic effects are mediated by glutamate imbalance, which is the principal excitatory neurotransmitter in the brain. Methamphetamine administration leads to the release of excessive glutamate into the extracellular space, which can trigger excitotoxicity [65,68].

Methamphetamine can also impact mitochondria in microglia and neurons, disrupting mitochondrial homeostasis, oxidative stress metabolism, and mitochondrial morphology. Disruption in oxidative metabolism can initiate neurodegeneration [69] and may lead to single- or double-strand nucleic acid breaks caused by reactive oxygen species contributing to persistent chromosomal alternations [70]. Such neurobiological cascades are associated with the “Warburg effect”, a distinct metabolic state in which the brain prioritizes glycolysis rather than oxidative phosphorylation for energy generation [71]. This state is characterized by increased glycolysis [72], acidification, less efficient energy availability, albeit quicker, and altered cell signaling [73]. These findings suggest that methamphetamine use disorder does not only lead to dopamine dysregulation but also signifies an altered brain state, consistent with what is observed in other degenerative central nervous system disorders. These neurobiological effects of methamphetamine may interact with genetic predispositions to increase vulnerability to methamphetamine use and other commonly comorbid disorders. Variations in genes related to dopamine receptors, transporters, and other neurobiological systems can affect how the brain responds to methamphetamine, which may explain why some individuals who use methamphetamine can transition to methamphetamine use disorder while others with similar exposure do not.

## 3. Genetics of Methamphetamine Use Disorder

Substance use disorders are known to be heritable, with an overall heritability estimated at 40%. However, the heritability specific to methamphetamine use disorder remains unknown due to the lack of family or twin studies [74]. To address this, researchers have used GWASs, which are performed to detect single-nucleotide polymorphisms (SNPs) in individuals with methamphetamine use disorder. SNPs are variations at a single position in the DNA sequence among individuals, and their detection is important because they can help identify genetic factors that contribute to disease susceptibility [75]. So far, five GWASs have been conducted on individuals with methamphetamine use disorder [17,18,19,20,76]. Uhl et al. (2008) [20] underscored the polygenic contributions to methamphetamine dependence, identifying overlapping genetic variants that also influence dependence on other addictive substances. Ikeda et al. (2013) [18] highlighted the shared genetic risk between methamphetamine-induced psychosis and schizophrenia, supporting a genetic overlap between these conditions. Zhang et al. (2020) [76] identified several sociodemographic and genetic risk factors for persistent methamphetamine-related psychiatric symptoms. However, none of these studies detected any significant genome-wide SNP associations with methamphetamine use disorder. Sun et al. (2021) [19] and Chang et al. (2022) [17] together identified associations between methamphetamine use disorder and SNPs in three specific genes with the following novel loci: *ANKS1B* rs2133896, *AGBL4* rs147247472, and *CTNNA2* rs1019686. However, all five studies were severely underpowered for GWASs [14], with sample sizes that were far too limited to detect reliable genome-wide findings compared to other similar polygenic complex psychiatric disorders [77,78,79]. The reason for these GWASs being underpowered may simply be that there are currently too few biological studies on people with methamphetamine use disorder for researchers to collect sufficient sample numbers to meet the stringent statistical thresholds required for GWASs [80]. To address the challenge of limited sample size, several potential solutions can be implemented, such as conducting multi-center studies to improve power [81] and leveraging biobanks to facilitate access to larger cohorts encompassing a variety of ethnic backgrounds [82].

Due to the existing GWASs being underpowered, researchers in methamphetamine use disorder are turning to meta-analytical findings from candidate gene studies as a more cost-effective alternative. Meta-analyses enable the combination of data from multiple studies, which enhances statistical power and increases the likelihood of detecting significant associations. Meta-analyses of candidate gene studies can provide insights by aggregating data from multiple smaller studies to assess reliability. Furthermore, candidate gene studies can identify genetic variations related to different aspects of methamphetamine use disorder, such as psychiatric symptoms and cognitive function, which is especially valuable in understanding complex disorders like methamphetamine use disorder. A meta-analysis [14] and an association study [83] from our laboratory identified several candidate genes with notable associations with methamphetamine use disorder and related cognitive function.

### 3.1. The Complex Relationship Between BDNF and Methamphetamine Use Disorder

Brain-derived neurotrophic factor (BDNF) is a member of the neurotrophin family and is expressed in various regions of the mammalian brain important for substance use disorders, such as the ventral tegmental area [84], hippocampus, nucleus accumbens, and frontal cortex [85]. BDNF is involved in the mechanisms underlying mood disorders and drug addiction, playing a crucial role in promoting neuronal growth and enhancing synaptic transmission [86]. For example, BDNF has been demonstrated to enhance the survival and protection of dopaminergic neurons in rodents with neurological symptoms [87] following methamphetamine administration [88].

The *BDNF* gene is located on chromosome 11p13 and contains a common and functional SNP, rs6265 (Val66Met), at codon 66. This G196A polymorphism results in a valine (Val)-to-methionine (Met) substitution, which affects the intracellular trafficking and activity-dependent secretion of BDNF [89]. Research has found that the valine (196G) allele is more common in people with methamphetamine dependence compared with methamphetamine naïve individuals [90]. Additionally, a meta-analysis showed that the *BDNF* rs6265 minor allele (Met) was associated with a decreased risk of methamphetamine use disorder and suggested that the Met allele may protect against methamphetamine’s rewarding effects by lowering BDNF protein levels [91]. In addition, the Met allele of the Val66Met polymorphism in the *BDNF* gene was associated with a lower severity of methamphetamine use in people with methamphetamine use disorder [83]. This polymorphism is located in the 5′pro-*BDNF* sequence, which encodes the precursor peptide (pro-BDNF) that ultimately forms the mature protein [92]. Considering the mechanism of action of BDNF, the Met/Met variant might slow the packaging and transport of BDNF, leading to reduced secretion and impairment of its function [93].

BDNF’s role in neurodevelopment may explain the contrasting finding that Met/Met and Val/Met carriers are linked to an earlier age of onset of methamphetamine use compared with Val/Val carriers [94]. However, findings of elevated BDNF levels in peripheral blood samples were largely consistent regardless of the age of people who used methamphetamine. For example, adolescents who used methamphetamine had higher BDNF levels compared with control adolescents [95]. Elevated levels of BDNF expression have been observed following methamphetamine use in adults, particularly in the acute phase [96], although no differences in *BDNF* mRNA levels between people with and without methamphetamine use disorder have been reported [83].

### 3.2. Reduced FAAH Function May Confer Susceptibility Toward Methamphetamine Use Disorder

The endocannabinoid system is one of the most important neural signaling pathways related to reward processing in substance use [97]. The fatty acid amide hydrolase (*FAAH*) gene encodes FAAH, an enzyme responsible for the breakdown of endogenous cannabinoids, like anandamide, which regulates mood, stress, and reward [98].

Studies have suggested a potential link between variations in the *FAAH* gene and susceptibility to substance use disorders, including methamphetamine use disorder, in various ethnic groups [99] and adolescents [100]. Importantly, the C385A variant (rs324420) of the *FAAH* gene has been associated with changes in endocannabinoid levels and increased sensitivity to reward-related stimuli. The C385A variant in the *FAAH* gene leads to a decrease in FAAH enzyme activity [101,102]. This reduction is due to the variant causing the enzyme to become more susceptible to proteolytic degradation, resulting in lower FAAH protein levels and activity. This ultimately leads to increased anandamide levels and endocannabinoid signaling, potentially elevating the dopamine release [101,102]. This variant has also been associated with a higher vulnerability to substance use disorders, including methamphetamine use disorder [103]. Multiple sources have examined the rs324420 variant of *FAAH* and demonstrated its association with methamphetamine dependence or use disorder [99,104], which has also been observed in a meta-analysis [14]. People with methamphetamine use disorder also showed lower levels of blood *FAAH* mRNA compared with controls who did not use methamphetamine, suggesting elevated anandamide levels may be associated with the disorder [83].

### 3.3. Reduced SLC18A1 and SLC18A2 Function May Confer Risk for Methamphetamine Use Disorder

Methamphetamine directly interacts with vesicular monoamine transporter (VMAT) proteins to play a crucial role in regulating monoamine transport from the cytosol to synaptic vesicles [45]. VMAT1 and VMAT2 are two distinct transporters that are encoded by the *SLC18A1* and *SLC18A2* genes, respectively [105]. Various polymorphisms in *SLC18A1* are associated with several neuropsychiatric disorders, including alcohol use disorder, schizophrenia, bipolar disorder, and major depressive disorder [46,106,107,108,109]. VMATs exchange monoamines with protons across the membrane of secretory and synaptic vesicles. They also mediate presynaptic monoaminergic vesicle transport in the amygdala and prefrontal brain regions related to emotion processing in response to environmental stimuli [110]. Although VMAT1 and VMAT2 are similar in structure, their properties differ based on VMAT1 exhibiting lower turnover and lower affinity to monoamines compared with VMAT2 [43,111]. Like *SLC18A1*, genetic variations in *SLC18A2* have been found in people with various neuropsychiatric and substance use disorders [112,113,114].

Despite the presence of several genetic variants of *SLC18A1* that have been reported in brain-related disorders, making it a potential candidate gene, research on VMAT1 remains sparse compared with VMAT2, for which a substantial amount of literature is available (reviewed in [115,116]). In a preliminary study, our laboratory identified that people with methamphetamine use disorder are ∼6 times more likely to carry the *SLC18A1* rs2270641 heterozygous genotype compared with controls who do not use methamphetamine [83], though this should be replicated in a larger sample size. This variant is also implicated in schizophrenia [106].

*SLC18A2* has known interactions with methamphetamine [20,116]. A study reported a 10% reduction in VMAT2 binding in people who use methamphetamine, with no correlation between VMAT2 expression and abstinence length, suggesting potentially irreversible damage from methamphetamine use [117]. *SLC18A2* genetic polymorphism rs363227 has been linked with reduced severity of methamphetamine use disorder [83]. The minor allele may be associated with increased VMAT2 function [118]. This observation is in agreement with the notion that individuals with the rs363227 wild-type allele may need to use higher doses of methamphetamine to achieve the rewarding effect of the drug compared to individuals with the minor allele [83].

## 4. Cognitive Deficits Associated with Methamphetamine Use

Genetic contribution toward methamphetamine use disorder may play a role in the cognitive dysfunction associated with the disorder. Cognition refers to the mental processes involved in acquiring, processing, and utilizing knowledge, covering functions such as perception, memory, attention, reasoning, and decision making [119]. While acute methamphetamine use can transiently enhance cognitive alertness and abilities, particularly attention [6], chronic use is associated with lasting brain dysfunction [11]. This ultimately results in sustained cognitive deficits, particularly impacting inhibition and decision-making tasks where the most significant impairments are observed [120,121]. Between 40% and 60% of people who use methamphetamine are estimated to experience cognitive impairments [122,123]. This cognitive profile is further supported by neuroimaging evidence, which reveals that chronic methamphetamine use alters medial frontal–striatal networks that are crucial for reward-based decisions and inhibitory control [11,124,125]. Chronic methamphetamine use is also associated with hypofrontality, a condition characterized by reduced activity in the frontal cortex, which is linked to deficits in learning, memory, attention, and impulse control [126]. Impairments in cognitive abilities can severely hinder daily living, resulting in significant consequences such as social isolation and unemployment [127]. This has major treatment implications given that the principal treatment is psychotherapy, which requires cognitive engagement [128]. Cognitive dysfunction associated with methamphetamine use disorder largely involves executive function [11] that has been associated with reactivity to methamphetamine-related cues [91] critically associated with relapse behaviors [129]. Therefore, these two cognitive aspects of methamphetamine use disorder are the focus of the present review.

### 4.1. Executive Functions

Executive functions are intricate cognitive processes primarily needed for maintaining focus and attention that include working memory, problem solving, reasoning, multitasking, and inhibition [130]. In people with methamphetamine use disorder, these processes are often impaired significantly, contributing to decreased inhibitory control, reduced cognitive flexibility, impaired working memory, and slower attention and processing speeds [11]. Deficits in executive functioning in methamphetamine use disorder are of particular importance in relation to diagnosis and treatment as these impairments are associated with poorer treatment outcomes and increased risk of relapse [131]. Executive functions play a critical role in the prognosis of treatment efficacy and in preventing relapse, suggesting that improvement of patients’ executive function may enhance the effectiveness of treatment [132].

#### 4.1.1. Inhibitory Control

Inhibitory and attentional control are particularly important in the context of methamphetamine use disorder, as impairments in these areas can lead to drug-seeking behavior and relapse [133], with these impairments being evident in the literature [120]. Among neuropsychological tests, inhibitory control can be tested using the Stroop task, which measures the ability to suppress irrelevant information [134]. In this task, participants are asked to name the ink color of words as quickly as possible, rather than reading the words themselves. For example, in a congruent trial, the word “red” is displayed in red ink, while in an incongruent trial, the word “red” may be displayed in blue ink [135]. The interference caused by the conflicting information (the word versus the color) requires participants to inhibit the automatic response of reading the word, thereby testing their ability to control attention and process conflicting information [136]. In some variations of the Stroop task (switching), participants may be required to switch between different rules or tasks (e.g., naming colors in one block and reading words in another). This requires adaptation of responses based on the current instructions [134]. A considerable amount of literature indicates that chronic methamphetamine use is associated with impaired Stroop task performance in both adolescents [121,137,138] and adults [13,139,140,141] compared with controls who do not use methamphetamine.

#### 4.1.2. Attention and Processing Speed

Chronic methamphetamine use is often linked to impaired sustained attention, as well as reduced accuracy in attention-demanding tasks, leading to challenges in maintaining goal-oriented behavior. A meta-analysis showed that chronic methamphetamine use is associated with various cognitive deficits, including information-processing speed and attention [142]. Neuropsychological assessments like the Trail Making Test Part A (TMT-A) and the symbol coding task are commonly employed to evaluate attention and processing speed [13]. The TMT-A is a timed test where participants are required to connect a sequence of numbered circles as quickly as possible, widely used for the evaluation of processing speed as well as other executive domains [143,144]. In the symbol coding task, participants match symbols to numbers or letters under a time constraint [145]. This task requires sustained attention and rapid processing speed and is particularly sensitive to cognitive slowing, a common deficit among individuals who use methamphetamine [146]. People with methamphetamine use disorder often exhibit diminished accuracy and slower response times in this task compared with the control group, reflecting impairments in sustained attention and cognitive processing speed [147].

#### 4.1.3. Working Memory

Deficits in working memory have been reportedly associated with both current [148] and previous [141] methamphetamine use. As working memory is essential for the temporary storage of information, its disruption can hinder daily functioning and pose a significant barrier to recovery for individuals using methamphetamine [149]. Working memory can be effectively assessed by the spatial span and digit span tasks, both of which target different aspects of working memory and are sensitive to the impairments frequently observed in this population [150]. The spatial span task evaluates visuospatial working memory by requiring test participants to remember and replicate a sequence of spatial locations, giving an overview of the ability to hold and manipulate spatial information [151,152]. People with methamphetamine use disorder often experience difficulties with spatial span tasks, showing decreased span capacity and an increased error rate, likely due to methamphetamine-related damage in brain regions crucial for spatial working memory relative to the controls [153]. The digit span task measures both the storage and manipulation of verbal working memory by requiring participants to repeat sequences of numbers both forward and backward [154]. Similar to the spatial span task, utilization of the digit span task in the cohort of people who use methamphetamine has reported poorer performance compared with healthy individuals, with length of abstinence being positively related to improved performance [155]. A meta-analysis on methamphetamine-related cognitive difficulties showed that people who use methamphetamine showed significantly worse working memory performance compared with controls, as assessed by the digit span and spatial span tasks [152], among others [120,156].

#### 4.1.4. Cognitive Flexibility

Cognitive flexibility is the mental ability to adapt to changing circumstances or environmental conditions, a key component of executive functioning [157]. In humans, executive functions comprise different forms of cognitive flexibility mediated by the prefrontal cortex [158], a region affected by methamphetamine use [159]. This flexibility in cognition allows individuals to switch between tasks, manage competing information, and modify strategies, which is often impaired by the neurotoxic effects of methamphetamine on the prefrontal cortex [160]. Cognitive flexibility can be assessed by a range of neuropsychological tests, including the Stroop switching task, which requires participants to identify the color of a word (which may be a conflicting color name) while occasionally switching between different rules or response sets [134,161]. Another commonly used assessment for cognitive flexibility is the Trail Making Test Part B (TMT-B) task, in which participants are required to alternate between letters and numbers in a sequence [144]. Research shows that individuals with methamphetamine use disorder exhibit significantly impaired cognitive flexibility, as demonstrated by poor performance on the Stroop switching task [162] and other similar assessments [163], compared with controls.

### 4.2. Cue Reactivity

Cue reactivity refers to the psychological and physiological responses of an individual to drug-related cues, such as images, videos, sounds, environmental stimuli, or drug paraphernalia. These cues can elicit conditioned responses, which are automatic reactions developed through repeated associations between a stimulus and a specific outcome. Examples include increased heart rate, altered brain activity, or sweating, reflecting the learned association between the cue and the drug’s effects [13,164]. These responses are attributed to Pavlovian conditioning, in which individuals associate certain stimuli related to the drug-taking experience with the drug’s availability and rewarding effects [165]. Cue reactivity is pivotal in understanding the processes of craving and relapse in substance use disorders. When exposed to drug-related cues, individuals can experience heightened craving [166]. Cue reactivity is aggravated by deficits in inhibitory control. Reactivity to the cues is predictive of the relapse risk, with a direct relation being observed between stronger reactivity and higher rates of relapse [164].

Cue reactivity is often measured with self-report in combination with skin conductance response (SCR). SCR reflects changes in sweat gland activity, which is often accompanied by a craving-like response or anticipation of substance use [167]. For the evaluation of chronic methamphetamine effects on cognitive functioning, cue reactivity along with SCR has emerged as a valuable tool for measuring participants’ arousal levels in response to drug-related cues [168]. In the first study to incorporate both control cues and control participants with subjective and objective measures, people with methamphetamine use disorder showed more SCR and self-reported craving for methamphetamine compared with control cues, whereas the methamphetamine-naive control group did not show differences in either of those measures to the different types of cues [13]. This finding indicates that cue reactivity in individuals with methamphetamine use disorder may be attributed to Pavlovian conditioning of the methamphetamine-taking experience. The paradigm’s novel design facilitated clear observation of methamphetamine cue reactivity in people with methamphetamine use disorder. In the study, control cues were specifically designed to ensure that the physiological and subjective responses observed were specific to methamphetamine-related stimuli. These control cues were images and videos of food-related items and activities. The rationale for using food-related cues was that both food consumption and methamphetamine use involve motor activities and share similar characteristics (e.g., object usage). This approach improved upon previous paradigms that have used either nature or sexual cues, which may either be too relaxing or too arousing even for control participants (reviewed in [164]). The results indicated that individuals with methamphetamine use disorder exhibited greater subjective reactivity and cue-specific physiological responses compared with controls. Furthermore, positive correlations were observed between physiological reactivity and the duration of methamphetamine use, indicating that objective cue reactivity measures can indicate meaningful information regarding methamphetamine use history [13].

## 5. Gene–Cognition Interplay

Recent research highlights an interplay between genetic factors and cognitive function in individuals with methamphetamine use disorder. Elevated BDNF levels, while crucial for brain function, were negatively correlated with the Neurocognitive Index—a composite measure of overall cognitive functioning— in adolescents with methamphetamine use disorder [95]. Specifically, higher BDNF levels were associated with lower scores on the Neurocognitive Index, indicating greater cognitive deficits. Additionally, the Neurocognitive Index was negatively associated with the duration of methamphetamine use, meaning that longer periods of use corresponded to lower cognitive functioning. These findings suggest a link between increased BDNF levels, prolonged drug use, and reduced cognitive performance [95]. However, in adults, inhibitory control and cognitive flexibility showed no association with *BDNF* mRNA levels [83]. *FAAH* gene expression also plays a role, with lower *FAAH* mRNA levels negatively correlating with inhibitory control performance [83]. Such findings suggest that increasing anandamide levels may be associated with reduced inhibitory control. Furthermore, a significant difference was observed in Stroop inhibition scores across *SLC18A2* rs363387 genotypes, with individuals carrying the wild-type genotype showing poorer performance than those with at least one minor allele. The wild-type genotype may be associated with reduced VMAT2 function [118], which suggests that VMAT2 levels are associated with inhibitory control. Additionally, higher peripheral *SLC18A2* mRNA levels were inversely associated with Stroop switching performance, suggesting that increased expression of *SLC18A2* corresponds to better cognitive flexibility [83].

Such gene–cognition interplay is important to understand methamphetamine use characteristics. For example, decreased cognitive flexibility in people who use methamphetamine is associated with a longer history of methamphetamine use [169], which suggests that the *BDNF, FAAH*, or *SLC18A2* genotypes may affect cognitive control when using methamphetamine. Alternatively, blood mRNA and/or protein levels of these genes may be affected by chronic methamphetamine use, which, in turn, impacts cognition.

**Figure 3:**
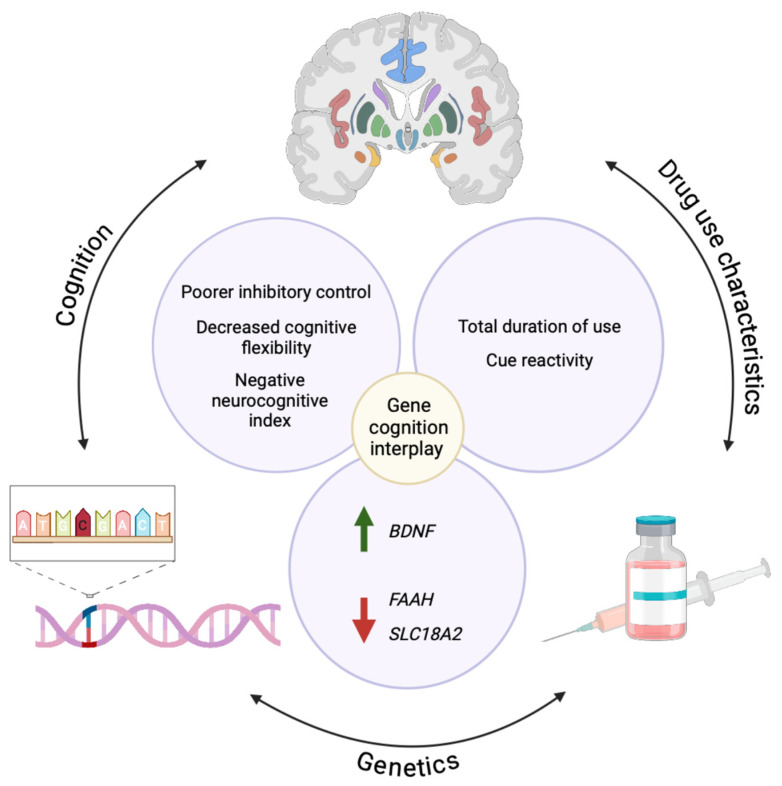
The interplay between genetics, cognition, and drug use characteristics in methamphetamine use disorder. There is emerging evidence of significant associations between *BDNF, FAAH,* and *SLC18A2* expression and cognitive performance that may be explained by the total duration of methamphetamine use and reactivity to methamphetamine-related cues.

In another example, heightened cue reactivity measured as self-reported craving in response to methamphetamine-related cues was negatively correlated with inhibitory control in people with methamphetamine use disorder [13]. In that study, more years of methamphetamine use correlated with stronger physiological cue reactivity, and people who used methamphetamine intravenously showed higher physiological cue reactivity than people who smoked, likely due to the greater bioavailability of injected methamphetamine. Together, these findings indicate that there may be a dose-related effect in Pavlovian conditioning of methamphetamine use, with more methamphetamine used for a longer period of time leading to stronger cue reactivity measured by sweating. Considering how cue reactivity is positively related to relapse and inversely related to inhibitory control, we hypothesize that genetic predisposition may affect inhibitory control to promote relapse to perpetuate chronic methamphetamine use. The intricate relationship between genes and cognitive deficits in methamphetamine use disorder is summarized in Figure 3.

## 6. Conclusions and Future Perspectives

This review highlights the recent evidence of interplay between genetic and cognitive factors in methamphetamine use disorder. Methamphetamine’s neurotoxic effects underscore the drug’s influence on cognition. Several biological factors and pathways have been associated with cognitive performance in people with methamphetamine use disorder, many of which have been explored in other disorders and may facilitate the development of novel therapeutic strategies. There are completed and ongoing clinical trials assessing pharmacotherapies to improve cognitive performance in people who use methamphetamine [170]. These pharmacotherapies typically target monoamine signaling, which works closely with the genes identified in the cognitive performance of people with methamphetamine use disorder in this review. Modulating the levels of key proteins may be achieved genetically [171] or pharmacologically [172] to enhance cognition to facilitate treatment and reduce relapse.

The best evidence-based behavioral therapies for methamphetamine use disorder include contingency management and cognitive behavioral therapy [128,173,174]. Contingency management reinforces sustained abstinence by providing immediate material rewards (e.g., vouchers) contingent on the provision of a negative drug urine test [175]. Cognitive behavioral therapy is a structured form of ‘talk therapy’ based on the principles of conditioning and learning. It helps individuals develop skills to reduce drug use, achieve initial abstinence, and prevent relapse in the long term [128,176]. However, these treatments are not without limitations. The effectiveness of contingency management can diminish once rewards are discontinued, leading to potential relapse, indicating that this type of therapy may not foster enduring behavioral changes [177]. For cognitive behavioral therapy, a limitation is the high dropout rate, as it requires a sustained commitment to long-term therapy [178]. Both contingency management and cognitive behavioral therapy rely on cognitive processes such as inhibitory control, as they require individuals to regulate impulses [179,180] and develop adaptive coping strategies to reduce methamphetamine use and relapse [181,182]. Thus, cognitive impairments associated with chronic methamphetamine use can reduce the ability to engage in and benefit from behavioral therapies [9,183]. No FDA-approved pharmacological treatments currently exist for methamphetamine use disorder or methamphetamine-induced neurotoxicity [8]. Together, understanding the biological basis of cognitive impairments in methamphetamine use disorder is critical for the development of new psychosocial therapies.

Knowledge of the genetic contribution of methamphetamine use disorder has promise to improve diagnostic precision and potentially lead to tailored treatment strategies. Genetic screening could help identify individuals at higher risk of developing dependence, facilitating early interventions. For example, individuals with polymorphisms in the *DRD2* (which encodes the dopamine receptor D2, influencing the brain’s reward system) [184] or *DAT1* [185] genes might benefit from preventive counseling or pharmacological approaches aimed at addressing dopamine dysregulation, as these genetic factors can influence the neurobiological response to methamphetamine and the likelihood of developing dependence. Moreover, pharmacogenomics, which examines how genetic factors influence drug responses, may be relevant for methamphetamine use disorder treatment. Variants in genes encoding drug-metabolizing enzymes, such as *CYP2D6*, can significantly affect the metabolism of medications used to manage methamphetamine withdrawal and dependence [186]. For instance, slower metabolizers might experience intensified drug effects or adverse reactions, requiring careful dosage adjustments [187,188]. Additionally, genetic variations in the *OPRM1* gene, which encodes the mu-opioid receptor, may influence individual responses to naltrexone [189,190], a medication currently under investigation for the treatment of methamphetamine use disorder [191].

Genetic research on methamphetamine use also has forensic, risk management, and ethical considerations. Forensic sciences can benefit from the exploration of the genetic underpinnings of methamphetamine-related deaths because genetic predispositions may interact with drug potency or poly-drug use to exacerbate methamphetamine’s toxic effects. For example, variations in genes involved in cardiac function, such as *SCN5A* [192], or in the metabolism of drugs, such as *CYP2D6* [193] and *CYP3A4* [194,195], can result in fatal arrhythmias or other adverse effects in people who use methamphetamine. Understanding these genetic factors is essential to inform people who use methamphetamine to prevent harm. However, the potential for genetic screening to diagnose methamphetamine use disorder may reveal sensitive information about an individual’s predisposition, raising concerns about privacy and the possibility of genetic discrimination. The Substance Abuse and Mental Health Services Administration (SAMHSA) advises against the routine use of genetic testing as the sole indicator of substance use disorder risk in clinical practice, emphasizing the need for comprehensive and multidimensional risk assessment [196]. Similarly, while cognitive training could help improve treatment outcomes, there is a risk of exacerbating social inequalities if access to such interventions is limited or unequal.

Lastly, there are critical areas for future research necessary to facilitate personalized medicine for methamphetamine use disorder. For example, understanding the age of onset of methamphetamine use and its relationship with genetic predisposition as well as drug use characteristics is particularly important for advancing our knowledge of the disorder’s trajectory [14,170]. Furthermore, some studies have not adequately distinguished between normative and problematic use, limiting the ability to draw conclusions about disorder progression. In addition, there are significant sex differences in patterns of use, clinical symptoms, and treatment responses [197]. For example, females report higher methamphetamine craving and mood disturbances compared with males, which may accelerate their transition from recreational use to drug dependence more quickly than males [198,199]. In contrast, males who use methamphetamine are more likely to engage in risk-taking behaviors compared with females, which can lead to higher rates of legal issues and physical health consequences [200]. Despite these differences, there is limited literature on the genetic and neurobiological mechanisms responsible for sex-specific responses to methamphetamine.

Taken together, methamphetamine use disorder is a complex disease associated with genes and cognitive impairments. Despite its prevalence and societal costs, effective therapies are lacking, which may be due to the severely limited understanding of how genes and cognition interact to perpetuate chronic methamphetamine use. Fortunately, recent studies have begun to unravel their potential relationship, providing a fresh outlook for understanding methamphetamine use disorder. We hope future research incorporates important aspects, such as the age of onset of use and sex specificity, to gain a deeper understanding of this complex disorder. Additionally, more studies and clinical trials including adolescents are essential to better understand the early impacts and progression of methamphetamine use disorder. Such a multidimensional approach will help to identify effective treatments and may even provide preventative strategies against casual use becoming disordered use.

## Data Availability

No new data were created or analyzed in this study. Data sharing is not applicable to this article.

## Abbreviations

The following abbreviations were used in this manuscript:

BDNF	Brain-derived neurotrophic factor
DAT	Dopamine transporter
FAAH	Fatty acid amide hydrolase
GWAS	Genome-wide association study
TLR4	Toll-like receptor 4
TMT-A	Trail Making Test Part A
TMT-B	Trail Making Test Part B
MD-2	Differentiation protein 2
SNPs	Single-nucleotide polymorphisms
VMAT1	Vesicular monoamine transporter 1
VMAT2	Vesicular monoamine transporter 2
